
JOURNAL INFORMATION
==============================
NLM Title Abbreviation: Innov Aging
Journal ID: Innov Aging
NLM Journal ID: 101703706
Journal ID: innovateage

Innovation in Aging

EISSN: 2399-5300
Publisher: Oxford University Press

ARTICLE INFORMATION
==============================
PMCID: PMC6840759
DOI: 10.1093/geroni/igz038.1055
Article ID: igz038.1055
Article version: 1
Subjects: Session 1355 (Poster), Family Caregiving I

TRANSITIONS OF FAMILY CAREGIVERS: A CONCEPTUAL FRAMEWORK FOR END-OF-LIFE CAREGIVING

Fox Aimee
Fruhauf Christine
Colorado State University , Fort Collins, Colorado, United States
Publication date: 2019 Nov
Electronic publication date: 2019 Nov 8
Volume: 3
Issue: Suppl 1
Issue title: Program Abstracts from the GSA 2019 Annual Scientific Meeting “Strength in Age—Harnessing the Power of Networks”
First page: S286
Last page: S286
Copyright: © The Author(s) 2019. Published by Oxford University Press on behalf of The Gerontological Society of America.
Copyright year: 2019
License: This is an Open Access article distributed under the terms of the Creative Commons Attribution License (http://creativecommons.org/licenses/by/4.0/), which permits unrestricted reuse, distribution, and reproduction in any medium, provided the original work is properly cited.
License URL: https://creativecommons.org/licenses/by/4.0/

Page count: 1

==============================
### TRANSITIONS OF FAMILY CAREGIVERS: A CONCEPTUAL FRAMEWORK FOR END-OF-LIFE CAREGIVING

Aimee Fox,[1] and Christine Fruhauf[1], *1. Colorado State University, Fort Collins, Colorado, United States*

Increased longevity and a growing aging population have led to rising numbers of informal, family caregivers. Compared to non-caregivers, caregivers are more stressed and depressed, and report lower levels of self-efficacy, physical health, and subjective well-being. Along the caregiving trajectory, end-of-life (EoL) care is emerging as a distinct phase that is not understood and warrants further research. This presentation will include an integrative literature review on EoL caregivers and propose a model for the transition to caregiving at the end-of-life. The review highlights the differences in strengths and needs of EoL caregivers when compared to non-EoL caregivers and age-related non-caregiving peers. For example, family members providing EoL care are more vulnerable to stress and psychological and physical strain, report low preparedness for EoL care, and often indicate they do not have the knowledge or skills needed for providing care at the EoL. The model for the transition to caregiving at the end-of-life presents new approaches to traditional stress and coping models and a conceptual framework for future EoL caregiving research and intervention efforts. The model highlights the need for expanding the theoretical foundations of EoL caregiving research and guides innovative methods of data collection and thoughtful approaches to the timing, recruitment, and utilization efforts of intervention programs. Ultimately, as researchers identify the needs of EoL caregivers, practitioners can improve caregivers health and well-being not only during this stage, but as they follow their own developmental path beyond loss and bereavement.
